# Supplementary material for: Pervasive Intestinal Carriage with Multiple Species of Extended Spectrum Cephalosporin-Resistant Enterobacterales in Children Admitted for Severe Acute Malnutrition at a Tertiary Hospital in Malawi
Source: medRxiv. 2025 May 31:2025.05.30.25328637. Preprint. [Version 1] doi: 10.1101/2025.05.30.25328637 (PMC12258776; doi:10.1101/2025.05.30.25328637)
Supplement: Supplement 1 [file NIHPP2025.05.30.25328637v1-supplement-1.pdf]

Supplemental Table 1

	Ampicillin	Ceftriaxone	Cefepime	Piperacillin-Tazobactam	Ertapenem	Gentamicin	Ciprofloxacin	Chloramphenicol
E. coli (n=137)	100	100	52.6	23.4	6.6	71.5	70.8	20.4
K. pneumoniae (n=108)	100	100	33.6	21.5	0.9	87.9	49.5	19.6
Enterobacter species (n=35)	100	100	15	10	5	75	70	10
K. oxytoca (n=3)	100	100	0	0	0	66.7	66.7	66.7
C. freundii (n=1)	100	100	0	0	0	100	100	100
E. bilingial (n=1)	100	100	100	100	0	100	100	0
A. baumannii (n=5)	100	100	20	40	100	20	20	100
P. aeruginosa (n=2)	100	100	0	0	50	50	0	100
**Total (n=293)**	**100**	**100**	**40.5**	**21.1**	**6.2**	**79.2**	**62.6**	**26.6**

medRxiv preprint doi: https://doi.org/10.1101/2025.05.30.25328637; this version posted May 31, 2025. The copyright holder for this preprint (which was not certified by peer review) is the author/funder, who has granted medRxiv a license to display the preprint in perpetuity.
It is made available under a CC-BY 4.0 International license .

Table S1: Antimicrobial non-susceptibility by species. Percent non-susceptibility reported.
